# Large Language Model–Powered Diagnostic Co-Pilot (“CapyEngine”) for Mental Disorders: Development, Evaluation, and Future Optimization Study

**DOI:** 10.2196/70017

**Published:** 2026-03-24

**Authors:** Liying Wang, Yunzhang Jiang

**Affiliations:** 1 Institute on Digital Health and Innovation, College of Nursing , Florida State University, 222 S Copeland St, Tallahassee, FL, 32306, United States, 1 (850) 644-3296; 2 Center of Population Sciences for Health Equity, College of Nursing, Florida State University, Talahassee, FL, United States; 3 Nexcuria Labs, LLC, Seattle, WA, United States

**Keywords:** large language model, LLM, mental disorders, diagnosis, ChatGPT-4, accuracy rate

## Abstract

**Background:**

Despite the growing potential of large language models (LLMs) in mental health services, evidence on its capabilities in diagnostic processes remains limited.

**Objective:**

This study described the development and evaluation of CapyEngine, an LLM-powered diagnostic tool designed to assist in the diagnosis of mental disorders.

**Methods:**

We developed and evaluated CapyEngine through 3 phases. In phase 1, we created a disorder and symptom database using *Diagnostic and Statistical Manual of Mental Disorders, Fifth Edition, Text Revision* (*DSM-5-TR*). We then designed and developed CapyEngine’s architecture using LLMs, embedding models, and vector searches. In phase 2, we conducted usability testing with mental health professionals (n=7). In phase 3, we compared CapyEngine’s diagnostic accuracy against ChatGPT-4o and clinicians using 35 standardized case scenario exam questions from psychiatry and clinical psychology board exams. Questions were input into CapyEngine, and the top 10 recommended diagnoses were obtained. ChatGPT-4o was prompted to provide the top 10 potential diagnoses for each question. Clinicians (n=3) received similar instructions to generate at least 10 potential diagnoses for each question. Responses were then analyzed to compare diagnostic accuracy of CapyEngine, ChatGPT-4o, and clinicians. Accuracy was measured by the percentage of questions where the correct answer was among the top 10 (least stringent), top 5, or top 1 (most stringent) results of the diagnosis list.

**Results:**

Preliminary user interview reflected high acceptability and feasibility of CapyEngine. Across diagnostic accuracy thresholds, ChatGPT-4o consistently outperformed both CapyEngine and clinicians in broader rankings (top 10 and top 5 benchmarks; all *P*<.03). Clinicians showed significantly higher accuracy than CapyEngine using the top 5 benchmark (odds ratio 0.26, 95% CI 0.09‐0.78; *P*=.02). For the top 1 benchmark, no significant differences were observed, where clinicians showed a borderline advantage over ChatGPT-4o (odds ratio 0.34, 95% CI 0.13‐0.91; *P*=.05). Regarding the range and slope of diagnostic accuracy decline across benchmarks (least to most stringent), CapyEngine showed the smallest decline (0.14) and flattest slope (−0.07), reflecting more consistent and constrained diagnostic ranking behavior as evaluation thresholds became more stringent. Clinicians exhibited a moderate decline (0.26), whereas ChatGPT-4o demonstrated a sharp decrease (0.69) in accuracy when only the top-ranked diagnosis was considered, consistent with broader diagnostic coverage at less stringent thresholds.

**Conclusions:**

Overall, ChatGPT-4o achieved the highest accuracy at less stringent benchmarks (top 10 and top 5), while clinician performance did not differ significantly from ChatGPT-4o in identifying the single most likely diagnosis. Although CapyEngine was less accurate overall, it exhibited more consistent and constrained diagnostic ranking across evaluation benchmarks, likely reflecting its DSM-5-TR–based, domain-specific design rather than broader diagnostic coverage. Nonetheless, CapyEngine shows promise as a tool to augment the mental health diagnostic process, and further research is needed to evaluate the risks and benefits of integrating artificial intelligence systems, such as CapyEngine, into clinical workflows.

## Introduction

Artificial intelligence (AI), since its birth, has been gradually shaping the landscape of health care [1]. AI is broadly defined as any technology that performs tasks that require cognitive capabilities that are usually considered unique to humans, such as reasoning, decision-making, and language [2]. Numerous studies on the use of AI to inform medical diagnostics, guide treatment planning, and assist in surgery have been conducted over the past decades [3,4]. The field is gradually uncovering the potential of AI in reducing human error, mitigating bias, increasing efficiency, easing clinician burden, and improving patient satisfaction [5]. Within psychiatry, machine learning (ML) models have been developed to predict mental health crises, mental illness onset, prognosis, inform selection of treatment, and predict relapse [6-9].

However, statistical ML models are limited in the following aspects, especially when it comes to psychiatry. First, training an ML model requires a large amount of structured data and preprocessing of the models, such as hyperparameter tuning, the construction of which is time-consuming and may be subject to arbitrary decision where human error and bias are inevitable [10]. Second, in psychiatry, the richness of textual data in an assessment or therapy session can be easily lost when only numerical or categorical data were extracted to input into the ML model [11]. Modeling multimodal data is still a technical challenge to adequately depict the complexity of mental disorders [12]. Third, the predictive capabilities of an ML model are highly restricted by the data it was trained on. Due to the lack of comprehensive data in mental health that covers all disorders across a diverse population, the existing ML models are only proficient in performing predictive tasks for a highly specified group of patients or disorders [13].

Generative artificial intelligence (genAI) models, the first of which was presented by OpenAI in 2022, have demonstrated the potential of artificial general intelligence with its ability to handle a diverse range of tasks characterized by some level of reasoning [14]. GenAI applications have been widely adopted in many fields, such as legal, criminal justice, higher education, and marketing. In health care, genAI models were found to be capable of passing the medical exams, outperforming physicians in providing empathetic and accurate responses to patient messages, and making medical diagnoses with simple prompting [15-17].

Reviews of genAI applications in mental health provided preliminary evidence on their capabilities in providing psychoeducation, coaching, and providing responses with empathy and accuracy [18,19]. However, evidence is limited in its ability to conduct assessment, generate accurate diagnosis, and demonstrate cultural sensitivity and competency in the process [20]. Mental disorder diagnosis requires comprehensive assessment and careful consideration of differential diagnosis. Depending on the level of training and complexity of the case, human bias and error could lead to misdiagnosis and delayed diagnosis and may lead to mistreatment and untreated mental health illnesses [1,21]. GenAI tools hold promise in mitigating these challenges in mental disorder diagnosis, with its broad knowledge base and its ability to process textual and multimodal data and the potential to be trained to learn domain-specific tasks. This study aims to (1) describe the development of a large language model (LLM)–powered diagnostic co-pilot, CapyEngine, (2) evaluate the diagnostic accuracy of CapyEngine, and (3) compare its performance with ChatGPT-4o and mental health professionals.

## Methods

In the following sections, we described the study activities in 3 phases from initial conceptualization and design of CapyEngine (phase 1), to usability testing of beta version (phase 2), to accuracy testing (phase 3).

### Phase 1. Database Building and Design of CapyEngine

We started with curating our own database that can be used by CapyEngine to generate accurate diagnoses. We decided to use the latest version of the *Diagnostic and Statistical Manual of Mental Disorders* (*Diagnostic and Statistical Manual of Mental Disorders, Fifth Edition, Text Revision* [*DSM-5-TR*]), issued by the American Psychiatric Association [22]. The clinical psychologist (first author) created a guideline for diagnosis and symptom extraction. The team reviewed the structure of *DSM-5-TR* to help the members understand the structure of the *DSM-5-TR* and the components of a mental disorder diagnosis. We allocated the sections in *DSM-5-TR* across a team of 5 research assistants to extract diagnoses and correspondent diagnostic criteria into a spreadsheet. The clinical psychologist on the team provided a training session before data extraction and ongoing support during the extraction. Specifically, for each disorder, we extracted data from the “Diagnostic Criteria,” “Diagnostic Features,” “Differential Diagnostic,” and “Comorbidity.” To ensure data accuracy, research assistants did not generate or interpret diagnostic content but copied *DSM-5-TR* text verbatim into predefined fields. Prior to data extraction, all research assistants completed a structured training session led by the clinical psychologist (first author), during which sample extractions were reviewed to ensure consistency and fidelity to the *DSM-5-TR* source. We then designed the front- and backend flow of CapyEngine to implement the user input and diagnosis output process. Details on software infrastructure and tools employed are listed in Table 1.

**Table 1.** Software infrastructure of CapyEngine.

Infrastructure	Description
Backend	Express.js: implements RESTful APIs^a^ for efficient handling of user requests and seamless interaction with MongoDB Atlas for data storage and retrieval Prompt-engineered AI^b^ models: leverages prompted LLM^c^ and embedding models on AWS^d^ GPU^e^ instances to enable accurate multisymptom searches, data extraction, and disorder matching
Cloud services and infrastructure	Heroku: ensures deployment and automatic scaling of backend services to maintain high availability and performance AWS GPU instances and inference endpoint: provides scalable and low-latency model serving for real-time AI inference, supporting intensive computation tasks required by prompt-engineered models
Database	MongoDB Atlas: manages and stores structured and unstructured data, including clinical notes, and supports hybrid search for efficient retrieval of diagnostic information
Frontend	React and Next.js: builds a responsive, dynamic user interface with enhanced performance and search engine optimization

a API: application programming interface.

b AI: artificial intelligence.

c LLM: large language model.

d AWS: Amazon Web Services.

e GPU: graphics processing unit.

### Phase 2. Usability Testing of the CapyEngine Beta Version

We developed an interview protocol to understand the routine diagnostic procedure, identify existing challenges and opportunities of incorporating CapyEngine in their workflow, and understand potential users’ concerns and perceived challenges (Multimedia Appendix 1). Participants were recruited through word of mouth to ensure credentials as mental health professionals. The testing sessions were conducted in-person or video conferencing, depending on the location and availability of the participants. Sessions lasted 1 to 1.5 h, including semistructured interviews and interactive sessions with CapyEngine. The lead author conducted all usability testing sessions with 7 participants who were mental health professionals (ie, psychiatrists, clinical psychologists, and clinical social workers) or master-level trainees (ie, master students of counseling studies). Data collection continued until no new usability themes or user concerns emerged across testing rounds, suggesting code and meaning saturation [23].

### Phase 3. Evaluation of Diagnostic Accuracy of CapyEngine and ChatGPT-4o

#### Evaluation Materials

We collected standardized case scenario multichoice questions (n=35) from United States Medical Licensing Examination (USMLE, n=30) and Examination for Professional Practice in Psychology (EPPP, n=5) to compare the diagnostic accuracy of CapyEngine, ChatGPT-4o, and mental health professionals [24,25]. USMLE is a 3-step examination required for medical licensure in the United States. It assesses a physician’s ability to apply knowledge, concepts, and principles that are important in health and disease management [26-33]. Given that the human evaluators were from the clinical psychology and counseling field, when selecting questions from USMLE, we focused on the ones that were in the field of psychiatry and screened out those questions that require extensive knowledge of medical or pharmacological knowledge to derive a diagnosis. EPPP is a licensing exam used by state and provincial psychology boards to assess a candidate’s competency in psychology [25,34]. It is required for licensure to practice psychology in the United States and Canada. The case scenarios of all included the age, sex, key symptoms, and symptom duration and varied from 25 to 200 words. We extracted the questions, multiple choice options, and the correct answer according to the answer key. See Table 2 for an example of USMLE and EPPP case scenario questions.

**Table 2.** Example case scenario questions from United States Medical Licensing Examination (USMLE) and Examination for Professional Practice in Psychology (EPP).

Question source	Example question	Multiple choice options	Correct response
USMLE	A previously healthy 33-year-old woman is brought to the emergency department by the Secret Service for stalking the president of the USA for 2 months. She claims to be married to the president’s twin brother and states that the president just had his twin kidnapped to avoid competition. She speaks rapidly and is difficult to interrupt. Her associations are often loose. She says, "I haven’t slept for days, but I won’t even try to sleep until my husband is rescued. God has been instructing me to take over the White House. I can’t wait to be reunited with my husband. I hear his voice telling me what to do.” When asked about drug use, she says she uses only natural substances. She refuses to permit blood or urine tests, saying, “I don’t have time to wait for the results.” Which of the following is the most likely diagnosis?	Bipolar disorder, manic, with psychotic features Brief psychotic disorder Delusional disorder Psychotic disorder due to general medical condition Schizophrenia	Bipolar disorder, manic, with psychotic features
EPPP	During your first session with a 38-year-old man, he tells you that he is homosexual and that he found out, several weeks ago, that his partner of 6 years is having an affair and is planning on moving out of their house. The man says that he is very nervous and anxious, that he wishes he wasn’t a homosexual, and that he lays in bed at night obsessing about his partner. His anxiety has affected his work: He is having trouble concentrating, is not getting along well with his coworkers, and has “called in sick” several times in the past 2 weeks. Based on these symptoms, the best diagnosis is:	Adjustment disorder with anxious mood Bereavement Ego-dystonic homosexuality PTSD^a^	Adjustment disorder with anxious mood

a PTSD: posttraumatic stress disorder.

#### Procedure of Evaluation

We first randomized the order of the exam questions using a random number generator. For ChatGPT-4o, we used the “Tell me the top ten potential diagnoses for the following case vignette” as the prompt to guide its behavior. We then input the questions one at a time following the order into CapyEngine and ChatGPT-4o. Each case was processed independently to minimize cumulative or cross-case memory effects. Members of the research team who were blind to the correct answer then extracted the top 10 results to each case scenario question from both platforms. Mental health professionals (n=3, ie, 2 clinical psychologists and 1 master-level counseling trainee) were recruited by word of mouth to answer the questions in the same order and asked to generate at least 10 diagnoses for each case scenario question.

Although the evaluation questions were presented in multiple-choice format, CapyEngine, ChatGPT-4o, and clinicians were prompted to generate open-ended lists of potential diagnoses. To evaluate accuracy, generated diagnoses were mapped to the multiple-choice answer options through a supervised adjudication process. One research assistant performed the initial mapping based on diagnostic equivalence (ie, exact matches or widely accepted synonymous diagnostic labels), which was then reviewed by a mental health professional. Any ambiguities or disagreements were discussed and resolved by consensus. No partial credit was assigned, and ambiguous or nonspecific responses were not counted as correct.

#### Measurements and Definitions

Accuracy is measured by the percentage of the questions where the correct diagnoses appeared in the top 10, top 5, and top 1 of the list of diagnoses generated by ChatGPT-4o, CapyEngine, or human clinicians. Top 10 was the least stringent accuracy benchmark, whereas top 1 was the most stringent.

### Analysis

For phase 2, we used rapid qualitative analysis to review the interview script and extract practical themes to guide the feature adjustment for CapyEngine [35]. For phase 3, descriptive analysis (percentages) was used to present the accuracy rate of CapyEngine, ChatGPT-4o, and clinicians. Fisher exact test was used to compare the frequency of correct or incorrect diagnoses occurring in the top 10, top 5, and top 1 diagnoses among CapyEngine, ChatGPT-4o, and clinicians. To evaluate the diagnostic consistency of CapyEngine, ChatGPT-4o, and clinicians, the range and slope of diagnostic accuracy decline were calculated as the evaluation criteria became more stringent.

### Ethical Considerations

This study was determined to be exempt by the Institutional Review Board at the University of Washington (Study ID: STUDY00005995). All participants provided informed consent prior to participation, including consent for audio recording and anonymized use of study data for research purposes. Participation was voluntary, and participants could withdraw at any time without penalty. No personally identifying information about participants was collected, and all responses were deidentified prior to analysis. Because the study involved clinicians providing diagnostic judgments rather than patient data, no patient information was collected or accessed during the study. Participants received US $400 compensation for approximately 4 hours spent on the evaluation.

## Results

### Phase 1. Database Building and Design of CapyEngine

The workflow of CapyEngine included the following components.

- Component 1. User input: the process begins with text entry by a mental health professional that contains mental disorder symptoms (in this study, the EPPP or USMLE test questions).
- Component 2. Information extraction layer: this module is GPT 3.5, whose primary function is to extract symptoms from unstructured text input (previous step) and build an input symptoms list. It identifies and isolates symptoms from input and transforms them into a list. This function was attained through prompt engineering.
- Component 3. Diagnostic factors vector embedding retrieval layer: (1 vector representation: Each extracted diagnostic factor from the input is transformed into a vector representation. This vectorization process converts qualitative data into a numerical format that allows for efficient comparison and searching within the database. (2) Vector search in the database: The vector representation of each diagnostic factor is then used to perform a vector search in a database of diagnostic factors extracted from *DSM-5-TR*. This search identifies 10 to 30 possible preset diagnostic factors that are most similar to the input diagnostic factor based on their vector proximity. These preset diagnostic factors are standardized symptoms or diagnostic criteria that are prestored in the database and served as benchmarks for comparison. 3) Candidate selection: the result of this vector search is a shortlist of candidate preset diagnostic factors that are potentially similar to the input diagnostic factor. This step ensures that only the most relevant diagnostic factors are considered for further analysis, significantly narrowing down the scope for the next stage.
- Component 4. LLM-based validation: For each input diagnostic factor and its 10 to 30 possible matching preset diagnostic factors obtained from the vector search, the LLM (GPT 3.5) performs a detailed, semantic-level analysis. Specifically, we used the LLM-as-judges framework, where prompt engineering was used to check negation or exclusion conditions to identify true matches between diagnostic factors extracted from input and the symptom database [36]. In this step, it creates a list of validated diagnostic factors. For each symptom extracted from input, the validation process determines the most likely matches by looking through the list of possible matches from the database.
- Component 5. Diagnostic-factor-to-mental-disorder match module: (1) Linking diagnostic factors to mental disorders: In the database, each preset diagnostic factor is linked to a corresponding mental disorder. Each diagnostic factor corresponded to one or more related mental disorders. (2 Matching process: After confirming the match between diagnostic factors from input and from the database, the system uses these links to quickly identify the mental disorders associated with these factors. This process generates a list of possible mental disorders for the clinician to further evaluate.
- Component 6. Output a list of diagnoses ranked in the order of likelihood of matching the symptom description in the user input.

### Phase 2. Usability Testing of the CapyEngine Beta Version

All users in the testing sessions (N=7) were mental health professionals at different stages of training, including counseling studies graduate trainee (n=1), clinical psychology graduate trainee (n=4), licensed clinical psychologist (n=1), and psychiatry resident (n=1). A few themes emerged in the interview and beta testing.

#### Theme 1. Pain Points in the Routine Diagnostic Process

Participants shared frustration around the cumbersome process in using DSM to make diagnosis. They found a lack of options other than flipping through PDFs, or using keyword-based search databases, and occasionally consulting Google search. This process is especially burdensome for participants in an inpatient setting, where they face a large volume of patients, required to write structured detailed notes in medical charts, synthesizing notes from interaction with patients with other lab or neuropsychological testing results. One participant who was in the inpatient psychiatry setting stated that he often relies on broad diagnostic categories to quickly rule in or rule out and then zoom in on a few specific categories to speed up the process. In addition, human error and blind spots were identified as a significant challenge. Participants recognized that their conceptualization and structure of the intake session (initial interview and diagnostic interview) might be biased by their own training and thus might miss edge cases and not be able to readily identify the symptoms in the disorders that they lacked training in.

#### Theme 2. Testing Results and Suggestions for Improvement

After interacting with CapyEngine, participants found it to be a potentially helpful tool in many aspects of their practice, including brainstorming diagnostic considerations when feeling stuck with a case, providing alternative perspectives on diagnosis to reduce bias, serving as a helpful prompt (or a knowledgeable supervisor) to think broadly about possible conditions, and using the tool for trainees in mental health to help them familiarize themselves with and think critically about diagnoses. For example, participants noted that the output was very thorough, even if it sometimes included surprising or less likely diagnoses. He appreciated being reminded of rare diagnoses that he might have missed. They overall found the color-coded matching of symptoms in the output, and the ranking system made it easier for them to see which symptoms in a disorder were aligned with the user input. They highlighted the potential for CapyEngine to significantly reduce the time they spent on making diagnoses from 1 hour to 2 minutes. They recommended adding features that can be used in real time during sessions, where clinicians can use generated differential diagnosis questions to guide intake sessions and conduct more comprehensive diagnostic assessments.

#### Theme 3. Perceived Concerns and Challenges

The participants expressed concerns and challenges in the development and usage of CapyEngine in their practice. Several participants emphasized the need for enhanced diagnostic accuracy, citing the low tolerance for error in a clinical setting. They noted that accurate diagnosis requires demographic and contextual factors to be taken into consideration. For example, age is an important demographic factor for diagnoses related to personality disorders, developmental disorders, and dementia-related disorders. Cultural considerations were also highlighted by the participants as they recognized that the AI model’s performance could be biased due to the database, algorithm, and implicit biased descriptions of patients by providers’ notes. In addition, mental health experiences and the expression of symptoms may differ across cultures. They highlighted the importance of AI tools, such as CapyEngine, to incorporate social and cultural understanding of mental health, such as cultural experiences and expressions of mental health symptoms. Participants also expressed concerns around the confidentiality of using AI in clinical settings, highlighting the need for secure data handling. One participant noted that the mistakes that AI tools make might be qualitatively different from those of human clinicians, which indicates that results from AI should be closely examined and evaluated and that AI and humans may complement each other in many aspects. Overall, participants found AI tools, such as CapyEngine, could be a valuable tool. However, they cautioned that such tools should not replace human judgment but rather serve as a supplementary device to optimize the diagnostic accuracy and performance of clinicians.

### Phase 3. Evaluation of Diagnostic Accuracy of CapyEngine and ChatGPT-4o

For the within top 10 category, ChatGPT-4o demonstrated significantly higher accuracy compared with both CapyEngine (35/35, 100% vs 22/35, 63%; odds ratio [OR] 0.00; *P*<.001) and clinicians (35/35, 100% vs 29/35, 83%; OR ∞; *P*=.03). No significant difference was observed between CapyEngine and clinicians (22/35, 63% vs 29/35, 83%; OR 0.35, 95% CI 0.11‐1.07; *P*=.11). In the top 5 category, ChatGPT-4o again outperformed both CapyEngine (35/35, 100 % vs 20/35, 57%; OR 0.00; *P*<.001) and clinicians (35/35, 100% vs 29/35, 83%; OR ∞; *P*=.03). Clinicians performed significantly better than CapyEngine (29/35, 83% vs 20/35, 57%; OR 0.26, 95% CI 0.09‐0.78; *P*=.02). For the top diagnosis outcome, no statistically significant differences were found between CapyEngine and ChatGPT-4o (17/35, 49% vs 11/35, 31%; OR 1.95, 95% CI 0.74‐5.14; *P*=.23) or between CapyEngine and clinicians (17/35, 49% vs 20/35, 57%; OR 0.67, 95% CI 0.26‐1.71; *P*=.48). However, clinicians showed a near-significant advantage over ChatGPT-4o (20/35, 57% vs 11/35, 31%; OR 0.34, 95% CI 0.13‐0.91; *P*=.05; Tables3  4).

**Table 3.** Accuracy rate comparison between CapyEngine, ChatGPT-4o, and clinicians.

Exam question count	CapyEngine			ChatGPT-4o			Clinicians		
	Within top 10	Within top 5	As top diagnosis	Within top 10	Within top 5	As top diagnosis	Within top 10	Within top 5	As top diagnosis
Number correct=m (number of 1 s)	22	20	17	35	35	11	29	29	20
Number correct=n (number of 0 s)	13	15	18	0	0	24	6	6	15
Accuracy rate=m^a^/N^b^×100% (%)	63	57	49	100	100	31	83	83	57

a m: number of questions that got correct answers.

b N: total number of questions.

**Table 4.** Comparison between CapyEngine vs ChatGPT-4o vs clinicians.

Comparison	Odds ratio (95% CI)	*P* value	Interpretation
Top 10			
CapyEngine vs ChatGPT-4o	0.00	<.001	Highly significant: ChatGPT-4o performs much better
CapyEngine vs clinicians	0.35 (0.11‐1.07)	.11	Not significant: trend toward clinicians being better
ChatGPT-4o vs clinicians	∞	.03	Significant: ChatGPT-4o performs better
Top 5			
CapyEngine vs ChatGPT4o	0.00	<.001	Highly significant — ChatGPT-4o performs better
CapyEngine vs clinicians	0.26 (0.09‐0.78)	.02	Significant—clinicians perform better
ChatGPT-4o vs Clinicians	∞	.03	Significant—ChatGPT-4o performs better
Top 1			
CapyEngine vs ChatGPT	1.95 (0.74‐5.14)	.23	Not significant
CapyEngine vs clinicians	0.67 (0.26‐1.71)	.48	Not significant
ChatGPT-4o vs clinicians	0.34 (0.13‐0.91)	.05	Borderline—clinicians may outperform ChatGPT4o

Considering the range and slope of diagnostic accuracy decline, CapyEngine demonstrated the greatest consistency in diagnostic accuracy across ranking thresholds, with performance declining modestly from 0.63 (top 10) to 0.49 (top 1; decline =0.14, slope=−0.07). In contrast, clinicians showed a larger decline from 0.83 to 0.57 (decline =0.26, slope=−0.13), and ChatGPT-4o exhibited the steepest drop from 1.00 to 0.31 (decline =0.69, slope=−0.34; Table 5).

**Table 5.** Range and slope of diagnostic accuracy decline.

System	Top 10	Top 5	Top 1	Decline (top10→top1)	Slope per level
CapyEngine	0.63	0.57	0.49	0.14	−0.07
ChatGPT-40	1.00	1.00	0.31	0.69	−0.34
Clinicians	0.83	0.83	0.57	0.26	−0.13

## Discussion

### Principal Findings

This study compared diagnostic performance among CapyEngine, ChatGPT, and human clinicians across multiple ranking thresholds. ChatGPT-4o demonstrated the highest overall diagnostic accuracy, particularly within the top 10 and top 5 predictions, while clinicians maintained competitive accuracy for top-ranked diagnoses. The high accuracy of ChatGPT-4o should be interpreted with caution as its pretraining data may have included USMLE and EPPP content, and high performance on these materials may reflect memorization rather than generalizable clinical reasoning. In contrast, CapyEngine showed lower absolute accuracy but exhibited the greatest consistency across ranking levels, with only modest declines in performance (0.63 to 0.49 from top 10 to top 1). This pattern suggests that CapyEngine provides more consistent and reproducible diagnostic reasoning, in contrast to the sharper variability seen in ChatGPT-4o and clinicians. Such consistency may reflect a more structured diagnostic process underlying the reasoning of CapyEngine, which could support clinical reliability and interpretability in real-world decision-support contexts.

The integration of AI-powered clinical support tools, such as CapyEngine, for mental health diagnosis holds great promise in addressing the current challenges faced by the field. Mental health diagnosis is notoriously time-consuming and prone to errors, with studies showing high rates of misdiagnosis for common conditions such as depression, obsessive-compulsive disorder, and bipolar disorder [37-39]. AI technologies have already demonstrated significant benefits in other medical specialties, improving diagnostic accuracy, increasing clinician productivity, and reducing error rates [40]. In mental health, AI algorithms have shown the ability to detect symptoms of various disorders with 63% to 92% accuracy by analyzing behavioral signals, voice recordings, and social media data. These tools can assist clinicians in making more informed decisions, potentially reducing the time to diagnosis and ensuring patients receive appropriate treatment faster [41]. For instance, AI-driven analysis of speech, text, and facial expressions has made significant strides in identifying early signs of mental health disorders. Given the global shortage of mental health professionals and the increasing demand for services, AI tools, such as Limbic Access, have shown promise in streamlining clinical assessments, which reduced clinician assessment time by an average of about 13 minutes per assessment while maintaining or improving clinical outcomes [42]. By leveraging AI’s capacity to process vast amounts of data and identify patterns, these tools could help redefine mental illnesses more objectively, identify them at earlier stages, and personalize treatments based on individual characteristics [43].

The performance of CapyEngine may be due to several reasons. CapyEngine primarily depends on a symptom-based dataset, which may not fully capture the complexity of the diagnostic considerations of mental health conditions. Many mental health disorders require consideration of nonsymptom-based factors to achieve a more accurate diagnosis. These factors include age, sex, the duration and progression of symptoms, functional impacts, substance usage, laboratory results, family history, stressors, and comorbid medical conditions [44]. These diagnostic factors improve the performance of CapyEngine on differential diagnosis. In addition, CapyEngine’s embedding model, NeuML/pubmedbert-base-embeddings, is designed for general biomedical text but is not specifically optimized for the nuances of mental health diagnostics [45]. While PubMedBERT provides robust embeddings for a wide range of biomedical concepts, it may not fully capture the subtle linguistic and contextual features unique to mental health. To address this limitation, there is a clear need to train an embedding model specifically on mental health data. Such models would be better suited to capture the complex semantic relationships that are crucial in mental disorder diagnosis. By utilizing a more specialized model, CapyEngine could greatly enhance its ability to retrieve more accurate diagnostic factors from the database.

An important interpretive consideration in this study is the confounding of system architecture and foundation model capability. CapyEngine employs a retrieval-augmented generation (RAG) pipeline built on GPT-3.5, whereas ChatGPT-4o represents a more capable, newer-generation model accessed via direct prompting. As a result, the differences in the observed performance cannot be unambiguously attributed to either architectural design or model capability alone. It is plausible that a portion of ChatGPT-4o’s advantage at broader thresholds reflects its greater parametric knowledge as a more advanced model, independent of the prompting strategy employed [46]. Conversely, CapyEngine’s most distinctive characteristic, its consistency across ranking thresholds and its smallest accuracy decline (0.14 vs 0.26 for clinicians and 0.69 for ChatGPT-4o), is more plausibly attributable to its structured, *DSM-5-TR*–anchored architecture, since a more capable base model would be expected to yield broader rather than more constrained diagnostic coverage. This pattern is consistent with the interpretation that CapyEngine’s reproducibility reflects an architectural advantage rather than a model-level one [47,48]. Future work should include ablation studies such as comparing CapyEngine directly against a GPT-3.5 baseline without the RAG pipeline or rebuilding CapyEngine on GPT-4o. This will more definitively isolate the contributions of system architecture from those of the underlying foundation model. It is important to note that CapyEngine’s flatter slope of accuracy decline may be partially a statistical artifact of its lower baseline performance, as a model with a lower accuracy ceiling has inherently less room to decline across thresholds. This limits the extent to which the flatter slope can be attributed solely to its structured RAG-based architecture and symptom-matching process.

### Limitation

This study has several limitations. First, the evaluation phase used standardized test questions with clinical case scenarios, which came with correct answers for verifying model output but may limit the external validity of the evaluation of model performance. The choice of using standardized case scenarios was due to the pilot nature of the evaluation and the primary goal of this study, which was to draw initial insights into the strengths and limitations of AI diagnostic tools compared to human clinicians. Nevertheless, clinical vignettes based on real patients or deidentified clinical notes may serve as future evaluation materials to enhance the external validity of the evaluation results. Second, the case scenario questions covered limited categories of DSM diagnoses. Future iterations of the study could stratify the number of questions based on DSM categories to more comprehensively assess the performance of AI diagnostic tools across a wide range of diagnoses. Third, participants in the usability evaluation were recruited through word of mouth, resulting in a convenience sample that may introduce selection bias and limit the generalizability of the usability findings, as participants were likely drawn from the authors’ immediate professional networks. Fourth, we only used simple prompt engineering in evaluating the diagnostic capabilities of GPT-4o. We are aware that the performance of GPT-4o may be improved with more sophisticated prompting methods such as chain-of-thought; however, the systematic evaluation of prompting strategies was beyond the scope of this study. Fifth, GPT-4o was likely trained on large-scale public internet data that may include USMLE and EPPP practice questions, introducing the possibility of data contamination that could partially explain its high performance at less stringent benchmarks and disadvantage retrieval-based systems, such as CapyEngine. Sixth, this study includes methodological limitations related to both models and human comparators. CapyEngine was built on GPT-3.5, whereas the comparator AI model was GPT-4o; thus, part of the observed performance gap may reflect differences in base model capability rather than system architecture and could potentially be reduced by upgrading CapyEngine’s foundation model. In addition, the human comparison group was small and heterogeneous (n=3), consisting of 2 licensed psychologists and 1 master-level trainee, and the inclusion of a trainee may have lowered the human performance benchmark and influenced comparisons between human clinicians and AI models. Finally, the adjudicators who mapped open-ended responses to the multiple-choice answer options were not blinded to the source of the responses (ie, CapyEngine, ChatGPT-4o, or clinician). Complete blinding was not feasible because the 3 sources produce structurally and stylistically distinct outputs, where CapyEngine generated a ranked structured list, ChatGPT-4o produced conversational prose, and clinicians wrote in their own natural language. This introduces the possibility of observer bias in the adjudication process, which should be considered when interpreting the accuracy comparisons across sources.

### Future Directions

We plan to enhance CapyEngine’s performance in several ways. First, we will incorporate a nonsymptom-based dataset. Specifically, CapyEngine will expand its dataset to include nonsymptom-based criteria. These include a range of factors, such as patient age, sex, symptom duration, functional impacts, substance use history, family background, stressors, and comorbidity. Integrating these variables in the system will provide a more holistic view of the patient’s information and condition, allowing for more comprehensive and precise diagnostics. Second, we will develop a mental health–specialized embedding model specifically trained on mental health data. We plan to apply a large corpus of psychiatric literature and recruit a team of clinicians to write and select high-quality data into our corpus. Such a specialized model will allow CapyEngine to interpret diagnostic factors more accurately in clinical notes, and thus, retrieve more relevant diagnostic factors from the database. Third, we will continue to keep collaborating closely with mental health professionals to ensure that CapyEngine remains aligned with the latest clinical guidelines and practices. Engaging with clinicians for feedback and insights will help refine the tool’s algorithms and ensure that it meets the practical needs of mental health care providers.

### Conclusions

This study evaluated the diagnostic performance and usability of an AI-assisted diagnostic tool, CapyEngine, in comparison with ChatGPT-4o and human clinicians. ChatGPT-4o demonstrated the highest diagnostic coverage within broad-ranking categories, though this finding should be interpreted cautiously, given the potential for data contamination from pretraining on publicly available USMLE and EPPP materials. Meanwhile, clinicians achieved superior accuracy for the top-ranked diagnosis. Although CapyEngine’s overall accuracy was lower, its diagnostic performance remained notably stable across ranking thresholds, reflecting consistent and reproducible reasoning. This consistency, combined with its structured and interpretable outputs, highlights CapyEngine’s potential as a reliable decision-support tool that prioritizes transparency and clinical safety. Usability testing further suggested that mental health professionals valued the model’s clarity and consistency, while recognizing the need for human oversight and contextual judgment. While the study’s small sample size and controlled scenarios limit generalizability, the findings underscore the importance of balancing accuracy, interpretability, and user trust when integrating AI systems into mental health diagnostic workflows. Future work should explore facilitators and barriers of these systems in more diverse and real-world clinical contexts.

## Supplementary material

10.2196/70017 Multimedia Appendix 1 Usability testing protocol.

## Abbreviations

AI: artificial intelligence
*DSM-5-TR*: *Diagnostic and Statistical Manual of Mental Disorders, Fifth Edition, Text Revision*
EPPP: Examination for Professional Practice in Psychology
genAI: generative artificial intelligence
LLM: large language model
ML: machine learning
OR: odds ratio
RAG: retrieval-augmented generation
USMLE: United States Medical Licensing Examination
