# Supplementary material for: Large Language Model–Powered Diagnostic Co-Pilot (“CapyEngine”) for Mental Disorders: Development, Evaluation, and Future Optimization Study
Source: JMIR AI. 2026 Mar 24;5:e70017. doi: 10.2196/70017 (PMC13012609; doi:10.2196/70017)
Supplement: Multimedia Appendix 1 [file ai-v5-e70017-s001.docx]

**Appendix. Usability testing protocol**

**Section 1: Routine diagnostic procedure: challenges and opportunities**

1. How do you currently diagnose mental disorders?
2. Prompt for: tools, methods, specific approach
3. Prompt for: technology-based tools
4. What are the challenges for you in making a diagnosis?
5. What have you tried that has resolved some of these challenges or made this process easier?
6. How long does it take for you to make a diagnosis?

**Section 2: AI assisted engine**

1. In what ways can an AI-assisted diagnosis engine make your life easier as a clinician?
2. What will the AI assisted diagnosis look like
3. Transcript to mapping diagnoses (This will entail you to submit your conversation transcript, or intake notes and the AI will give you a diagnosis accordingly.)

ii. LLM-conversation based (This will entail you to have a conversation with the AI, including clarifying questions, in order for the AI to gain an understanding of the patient’s situation. (Similar to the way we use ChatGPT)

(1) Or both?

2. What kinds of output delivered by the tool do you think might be helpful to your diagnoses process? (possible disorder name, related symptoms, analytical report, etc.)

3. How would you use the AI-assisted diagnosis tool in your current workflow?

**Section 3: Concerns and potential challenges**

1. What concerns do you have about AI-assisted diagnosis tool for therapists with varying levels of familiarity with technology
2. What do you think about the accuracy and reliability of diagnoses made with the assistance of AI? And why？
3. How would you like the system to handle cases where the AI's diagnosis differs from your professional judgment?
4. Do you believe an AI-assisted tool could impact the therapist-patient relationship? If so, how?
